# *Scolopendra subspinipes mutilans* Extract Suppresses Inflammatory and Neuropathic Pain* In Vitro* and* In Vivo*

**DOI:** 10.1155/2018/5057372

**Published:** 2018-12-17

**Authors:** Lakkyong Hwang, Il-Gyu Ko, Jun-Jang Jin, Sang-Hoon Kim, Chang-Ju Kim, Jung Won Jeon, Jin Hee Han

**Affiliations:** ^1^Department of Physiology, College of Medicine, Kyung Hee University, Seoul 02447, Republic of Korea; ^2^Kohwang Medical Research Institute, College of Medicine, Kyung Hee University, Seoul 02447, Republic of Korea; ^3^Department of Internal Medicine, Kyung Hee University Hospital at Gangdong, College of Medicine, Kyung Hee University, Seoul 05278, Republic of Korea; ^4^Department of Anesthesiology and Pain Medicine, College of Medicine, Kyung Hee University, Seoul 02447, Republic of Korea

## Abstract

**Background:**

Sciatic nerve injury develops from a variety of pathological causes, including traumatic injury and neuroinflammatory disorders, which are accompanied by pathological changes that have a critical impact on neuropathic pain and locomotor activity. Extracts of* Scolopendra subspinipes mutilans *(SSM) are used in traditional medicine for the treatment of a wide range of neuropathic diseases, including lower back pain, peripheral neuropathy, and sciatic nerve injury. Although SSM shows anti-inflammatory, antibacterial, and anticonvulsant activities, its diverse mechanisms of action remain unclear. Thus, the present study examined the effects of SSM* in vitro* and* in vivo*.

**Methods:**

To estimate the anti-inflammatory effects of SSM, inflammatory conditions were induced using lipopolysaccharide (LPS) in RAW 264.7 cells, and inflammatory-related factors were evaluated by enzyme-linked immunosorbent assay (ELISA) and western blotting analyses. Sciatic nerve crush injury (SNCI) was induced in rats using a surgical clip instrument. The effects of SSM in the SNCI model were evaluated in behavioral tests by calculating the sciatic functional index (SFI) and measuring thermal hyperalgesia sensitivity and by monitoring inflammatory factors expression in western blotting analyses.

**Results:**

We observed the anti-inflammatory effects of SSM treatment both in vitro and in vivo. The PGE_2_ and NO production were suppressed by SSM. Protein analyses indicated that expression of NF-*κ*B and degradation of I*κ*B*α* were suppressed by SSM treatment. In addition, the levels of iNOS, TNF-*α*, IL-6, and COX-2 expression were reduced by SSM treatment in RAW 264.7 cells and in the SNCI-induced animals. In behavioral studies, SSM treatment enhanced the SFI and improved the thermal sensitivity test results.

**Conclusions:**

Our results suggest that SSM suppresses the production of inflammatory factors via the NF-*κ*B pathway and accelerates the morphological and functional recovery of the peripheral nervous system. Hence, SSM may be a useful therapeutic candidate for treatment of neuropathic pain diseases.

## 1. Introduction

Sciatic nerve injury occurs from various etiological causes, such as neural pressure, bone fracture, inflammatory disorder, and surgical injury that lead to neuropathic pain. The pain associated with the sciatic nerve injury can be a pain to the thigh or leg from the hip down and may be accompanied by pain in the feet and toes. Moreover, sciatic nerve injury patients with neuropathic pain have lower quality of life than other chronic pain. One of the sciatic nerve injury symptoms is accompanied by neural inflammatory response [1]. In previous studies, sciatic nerve injury has been reported to mediate various cytokines such as tumor necrosis factor alpha (TNF-*α*), interleukin-1 beta (IL-1*β*), and interleukin-10 (IL-10) [2].

Various lesions of sciatic nerve injury occur during the inflammation process, and the expression of inflammatory cytokines, chemokines, and other mediators in inflammation increases [3]. Moreover, pathophysiological features of inflammation involve that a various inflammatory mediators such as nitric oxide (NO), prostaglandin E2 (PGE_2_) mediators, and cytokines were produced by neuronal inflammatory responses [4, 5].

Classically, the inflammatory mediators are regulated primarily at the level of mRNA expression via the involvement of transcription factor such as nuclear transcription factor (NF-*κ*B) and inhibitor of kappa B alpha (I*κ*B*α*) in macrophages [6, 7]. Furthermore, these processes are subsequently known to induce the transcription of proinflammatory factors such as inducible nitric oxide synthase (iNOS), TNF-*α*, and cyclooxygenase-2 (COX-2) after nerve injury [3, 7].

A natural extract,* Scolopendra subspinipes mutilans* (SSM), is a stable extract that eliminates toxic components and is used in traditional medicine to treat neuroinflammatory diseases [8]. There are a few studies on the metabolites by SSM, where the quinoline alkaloids are characterized as representative secondary metabolites from SSM. Furthermore, research into SSM has been shown to consist of peptides with potential to be developed as effective anti-inflammatory analgesics [9]. In Korea, China, and other Asian regions, SSM natural extracts have been used to treat a variety of diseases such as neurodegenerative diseases, neuropathic pain, and inflammatory diseases. In particular, SSM has been used for the treatment of patients with sciatic nerve injuries accompanied by neuroinflammation, but the therapeutic molecular mechanism of SSM is not yet clear. In previous studies, the pharmacological properties of SSM have been reported to exhibit anti-inflammatory, antibiotic, and analgesic effects [10]. Recent studies have reported that NF-*κ*B or mitogen-activated protein kinase (MAPK) pathway is activated in LPS-induced inflammation models in macrophage, and sciatic nerves in injury models have also been shown to activate inflammatory responses through the NF-*κ*B pathway [6, 11]. In addition,* Scolopendra* mixture was revealed to modulate the lymphocytes, downregulate cytokines, and upregulate IL-4, IL-10 on the rheumatoid arthritis induced inflammatory animal model [12].

Hence, we examined whether SSM treatment affects the expression of inflammatory mediators induced by LPS in RAW 264.7 cells. In addition, in vivo studies evaluated the efficacy of SSM treatment of functional recovery, pain severity, and inflammatory mediators in sciatic nerve crush injury (SNCI) of rats.

## 2. Materials and Methods

### 2.1. Extraction of SSM

The SSM used in this study was obtained from the Korean Pharmacopuncture Institute (Seoul, Korea). Extraction of SSM proceeded according to the study of Lim et al. [13] and the method provided by the Korean Pharmacopuncture Institute. Briefly, dried weight of 128.6 g SSM powder is stirred with 1000 ml of distilled water for 3 h. After precipitation in the refrigeration, the extraction solution is filtered and the filtrate is concentrated under reduced pressure at 70°C for 3 h using a rotary vacuum evaporator. The concentrated amount is adjusted to 90%, 80%, and 70% alcohol sequentially, followed by stirring for 1 h, followed by filtration and concentration. The whole amount is filtered and freeze-dried for 200 h in a freezing dryer. The yield of extract was 9.88 g (yield: 7.68%).

### 2.2. Cell Culture and Viability Analyses

The RAW 264.7 cell line derived from mouse macrophage was plated at a density of 2×10^4^ cells/cm^2^ on 100 mm culture dish. Cells were maintained in Dulbecco's modified Eagle's medium (DMEM; GIBCO, Carlsbad, CA, USA) supplemented with 10% heat-inactivated fetal bovine serum (FBS), 100 *μ*g/mL streptomycin, and 100 IU/mL penicillin (Lonza, Walkersville, MD, USA). The cells were cultured at 37°C in a humidified condition with 5% CO_2_. When plates reached 80–90% confluence, cells were scraped into fresh medium. To analyze viability, RAW 264.7 cells were plated at a density of 1×10^4^ cells/well into 96-well plates containing 100 *μ*L medium and incubated for 12 h. RAW 264.7 cells were preinduced with 1 *μ*g/mL LPS (*Escherichia coli* serotype 026:B6; Sigma, St. Louis, MO, USA) for 1 h and treated with media containing various concentrations (0.01 *μ*g/mL, 0.1 *μ*g/mL, 1 *μ*g/mL, 10 *μ*g/mL, and 100 *μ*g/mL) of SSM for 24 h. Subsequently, 3-(4,5-dimethylthiazol-2-yl)-2,5-diphenyltetrazolium bromide (MTT; Sigma) was added to a final concentration of 0.5 mg/mL, and cells were incubated for 1 h in a humidified incubator. The medium was removed, the formazan precipitate was solubilized in DMSO, and the absorbance was measured at 570 nm using a microplate reader (Bio-Rad, Hercules, CA, USA).

### 2.3. Measurement of NO and PGE_*2*_

RAW 264.7 cells were plated at a density of 1×10^5^ cells/well in 24-well plates. The cells were preinduced with 1 *μ*g/mL LPS for 1 h and treated with media containing various concentrations (0.1 *μ*g/mL, 1 *μ*g/mL, and 10 *μ*g/mL) of SSM for 24 h. Cell culture supernatants were collected for ELISA to measure NO synthesis and PGE_2_ concentration. Measurement of NO production was performed using a commercially available NO detection kit (iNtRON, Sungnam, Korea) in accordance with the manufacturer's instructions. Briefly, 100 *μ*L supernatant and 50 *μ*L N1 buffer were added to each well, followed by incubation at room temperature for 10 min. Then N2 buffer was added and reacted at room temperature for 10 min. The absorbance of each well was measured at a wavelength of 540 nm on a microplate reader (Bio-Rad). PGE_2_ synthesis was measured using a commercially available PGE_2_ competitive enzyme immunoassay kit (Amersham, Piscataway, NJ, USA) according to the manufacturer's instructions. Briefly, 100 *μ*L of the medium and standards were added to different wells on plates coated with goat-anti-mouse IgG, which were included in the kit. Mouse anti-PGE_2_ antibody and peroxidase-conjugated PGE_2_ were added to each well, and the plates were reacted at room temperature with agitation for 2 h. The wells were drained and washed, and then 3,3′5,5′-tetramethylbenzidine hydrogen peroxide solution was added. The plates were incubated at room temperature with agitation, and the reaction was stopped after 30 min by adding H_2_SO_4_. The absorbance at 450 nm was determined on a microplate reader (Bio-Rad).

### 2.4. Western Blot Analysis of Inflammatory Factor in RAW 264.7 Cells

To detect inflammatory mediator protein expression, cells were pretreated with 1 *μ*g/mL LPS for 1 h and then treated with various concentrations of SSM for 24 h. Incubated RAW 264.7 cells were collected in chilled phosphate buffered saline (PBS) and centrifuged at 13200 rpm for 10 min at 4°C, and then the supernatant was removed. Protein lysate was extracted according to a previously described method [14]. Cell pellets were gently homogenized in RIPA buffer consisting of 20 mM Tris-HCl (pH 7.5), 150 mM NaCl, 1 mM Na_2_EDTA, 1 mM EGTA, 1% Nonidet P-40, 1% sodium deoxycholate, 2.5 mM Na_4_P_2_O_7_, 1 mM *β*-glycerophosphate, 1 mM Na_3_VO_4_, 1 *μ*g/mL leupeptin (Cell Signaling Technology, Danvers, MA, USA), and 1 mM PMSF (Sigma) and incubated for 20 min at 4°C. Collected cells were centrifuged at 13200 rpm for 20 min at 4°C, and supernatants were collected. Protein content was measured using a Bio-Rad colorimetric assay kit (Bio-Rad). NF-*κ*B and I*κ*B*α* in the cells were determined using a Nuclear/Cytosol fractionation kit (BioVision, Mountain View, CA, USA) according to the manufacturer's instructions. Cell lysates were separated by 12% SDS-PAGE gel and electrotransferred onto nitrocellulose membranes (Amersham). Membranes were agitated in 5% skim-milk blocking buffer for 1 h and reacted at 4°C overnight with the primary antibody: mouse anti-*β*-actin or rabbit anti-iNOS, anti-NF-*κ*B, anti-I*κ*B*α* or goat anti-COX-2, anti-TNF-*α*, and anti-IL-6 (Santa Cruz Biotechnology, Santa Cruz, CA, USA). Blots were developed using HRP-conjugated IgG and an enhanced chemiluminescence detection kit (Bio-Rad). Bands were quantified using Molecular Analyst™ version 1.4.1 (Bio-Rad).

### 2.5. Experimental Animal Procedures and Drug Treatments

**Figure 1:**
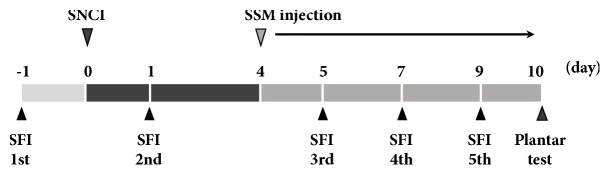
Experimental schedules.

Adult male Sprague-Dawley rats weighing 200 ± 5 g (8 weeks old) were randomly divided into five groups (*n* = 10 in each group): Sham operation group, SNCI-induced group, SNCI-induced and 0.1 g/kg SSM-treated group, SNCI-induced and 1 g/kg SSM-treated group, and SNCI-induced and 10 g/kg SSM-treated group. SNCI was induced according to a previously described surgical procedure [15]. Briefly, the right sciatic nerve was exposed by incision of the gluteal muscle with the rat under anesthesia with 50 mg/kg zolazepam and tiletamine mixture (Zoletil 50®; Virbac Laboratories, Carros, France). The sciatic nerve was carefully exposed and crushed for 30 sec using a surgical clip (Pressure: 125 g; Fine Science Tools Inc., San Francisco, CA, USA) placed between the sciatic notch and the point of trifurcation. After injury, the surgical wound was sutured and the animal was allowed to recover. Rats in the sham operation group underwent sciatic nerve exposure but the nerves were not crushed. For rats in the SSM-treated groups, the appropriate dosage of SSM (with 200 *μ*L 0.9% normal saline) was injected at the greater trochanter and anterior superior iliac spine in the hind leg at the midpoint of the connection once a day for 7 consecutive days, starting 3 days after surgery. For the determination of effective dosage of SSM, our preliminary experimental results and previous studies were considered [16, 17]. Therefore, we used a dosage of 0.1, 1, and 10 g/kg SSM in this study. All experimental schedules are shown in Figure 1. All procedures were performed in accordance with the Guidelines for the Care and Use of Animals approved by the National Institutes of Health Council for the Management and Use of Laboratory Animals. The experimental design was approved by the Institutional Care and Use Committee of Kyung Hee University (KHUASP[SE]-17-094).

### 2.6. Evaluation of Sciatic Function Index and Thermal Hyperalgesia in the SNCI of Rats

The functional recovery rate after sciatic injury was analyzed by walking tract assessment, which can be quantified with the sciatic function index (SFI). Walking analyses were performed 1 day before and 1, 5, 7, and 9 days after surgery. To evaluate the SFI, animals underwent conditioning trials in a 10 × 100 cm wooden track with a dark box at one end. The hind feet of rats were dipped in colored ink and the animals walked on white paper-covered tracks to leave footprints. The following parameters were obtained from the footprints: distance from the second to the fourth toe (intermediary toe spread, IT); distance from the heel to the top of the third toe (print length, PL); and distance between the first and the fifth toe (toe spread, TS). These measurements were made from both the nonoperated foot (NIT, NPL, and NTS) and the operated experimental foot (EIT, EPL, and ETS). These SFI parameters were applied by Bain et al. [18]. SFI index was according to a previously described method [19]. Interpolation of identical values of IT, PL, and TS from the right and left hind feet results in an SFI value close to 0 in normal rats, whereas an SFI value of –100 represents impairment. Thermal hyperalgesia was measured 10 days after surgery using a plantar test analgesia meter (Ugo Basile, Comerio-Varese, Italy). Briefly, the animals were placed into a plastic box and allowed to acclimatize to the testing space for at least 5 min before initiation of the behavioral test. After acclimation, radiant heat was applied to the floor of the hind paw on the east until the rat pulled out of the foot. Photoelectric cells automatically shut off the heat source when reflected rays are blocked. Intensity was set to 40 mW/cm^2^ low power with a heating rate of 1°C/sec to induce activation of unmyelinated fibers. The cut-off time was set as 35 sec based on the latency in the sham operation group.

### 2.7. Preparation of Tissue

Animals were sacrificed after the behavioral test for immunohistochemistry. The sciatic nerve was collected according to a previously described method [20]. The sciatic nerves were dissected and immediately immersed in a solution consisting of 4% paraformaldehyde in 100 mM PBS overnight. For cryoprotection, fixed tissue was immersed in a solution consisting of 30% sucrose in PBS. Longitudinal sections 20 *μ*m thick were cut using a cryostat (Leica, Nussloch, Germany). The tissue slides were incubated overnight at 4°C with primary antibody to neurofilament protein (NF-200; Sigma). After washing, the slides were incubated with secondary FITC anti-mouse IgG (Jackson ImmunoResearch Laboratories, West Grove, PA, USA) at room temperature. Tissues were mounted, and images were acquired using a Nikon Eclipse 50i (Nikon Inc., Melville, NY, USA) fluorescence microscope (100× to 400×). Analyses of individual sections used in the final quantification were performed using Image-Pro Plus software (Media Cybernetics Inc., Silver Spring, MD, USA).

### 2.8. Western Blot Analyses of Rats with Sciatic Nerve Crush Injury

The dissected sciatic nerves were homogenized on ice and lysed in RIPA buffer consisting of 20 mM Tris-HCl (pH 7.5), 150 mM NaCl, 1 mM Na_2_EDTA, 1 mM EGTA, 1% Nonidet P-40, 1% sodium deoxycholate, 2.5 mM Na_4_P_2_O_7_, 1 mM *β*-glycerophosphate, 1 mM Na_3_VO_4_, 1 *μ*g/mL leupeptin (Cell Signaling Technology), and 1 mM PMSF (Sigma). Aliquots of 30 *μ*g protein lysate were subjected to western blotting analyses by conventional SDS-PAGE with an electrophoresis apparatus (Bio-Rad). The membranes were immersed in blocking buffer for 1 h and reacted at 4°C overnight with primary antibody: mouse anti-*β*-actin, rabbit anti-iNOS, goat anti-COX-2, anti-TNF-*α*, and anti-IL-6 (1:1000; Santa Cruz Biotechnology). Blots were developed using HRP-conjugated IgG and an enhanced chemiluminescence detection kit (Bio-Rad). Bands were quantified using Molecular Analyst™ version 1.4.1 (Bio-Rad).

### 2.9. Statistical Analyses

Statistical analyses were performed using one-way ANOVA followed by Duncan's* post hoc* test using SPSS software (ver. 23.0; SPSS, Chicago, IL, USA), and the results are expressed as the means ± standard errors of the mean (SEMs). In all analyses, P<0.05 was taken to indicate statistical significance.

## 3. Results

### 3.1. Effects of SSM on Cell Viability, NO Synthesis, and PGE_*2*_ Production Following LPS Induction in RAW 264.7 Cells

**Figure 2:**
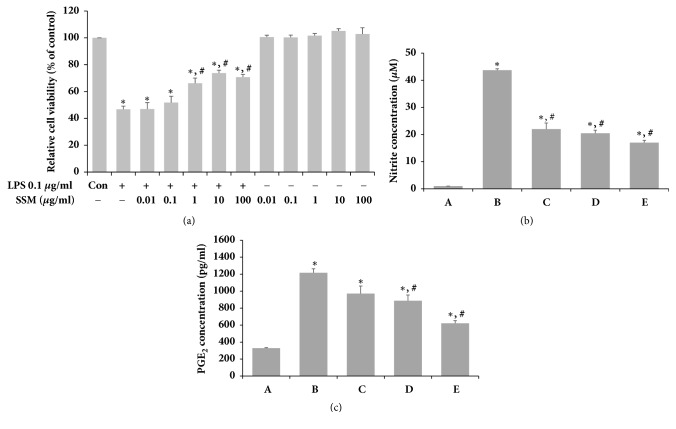
*Effects of SSM on LPS-induced cell viability, NO, and PGE*_*2*_ *in RAW 264.7 cells. *RAW 264.7 cells were induced with 1 *μ*g/mL LPS and various concentrations of SSM or with various concentrations of SSM alone for 24 h. (a) The cells were stained using the MTT method. (b) The production of NO in the culture medium was analyzed with a commercial NO detection kit. (c) The PGE_2_ contents in the culture medium were analyzed using a competitive enzyme immunoassay. (A) Control group, (B) 1 *μ*g/mL LPS-administered group, (C) 1 *μ*g/mL LPS-administered and 0.1 *μ*g/mL SSM-treated group, (D) 1 *μ*g/mL LPS-administered and 1 *μ*g/mL SSM-treated group, and (E) 1 *μ*g/mL LPS-administered and 10 *μ*g/mL SSM-treated group. The results are presented as the means ± standard errors of the mean (SEMs). ^*∗*^P<0.05 compared to the control group. ^#^P<0.05 compared to the LPS-induced group.

To determine the potential cytotoxicity of single SSM treatment, the viability of RAW 264.7 cells was measured using the MTT assay. SSM had no cytotoxic effects at concentrations from 0.01 *μ*g/mL to 100 *μ*g/mL (Figure 2(a)). In addition, it significantly increased cell viability in a dose-dependent manner after LPS administration. Based on these results, SSM was used at concentrations of 0.1 *μ*g/mL to 10 *μ*g/mL in subsequent experiments. The levels of NO and PGE_2_ production were determined by ELISA (Figures 2(b) and 2(c)). Induction of inflammatory conditions using LPS increased NO and PGE_2_ concentrations in RAW 264.7 cells. However, SSM treatment significantly suppressed NO and PGE_2_ expression under LPS-induced inflammatory conditions in a dose-dependent manner.

### 3.2. Effects of SSM on LPS-Induced Protein Expression of TNF-*α*, IL-6, iNOS, and COX-2

**Figure 3:**
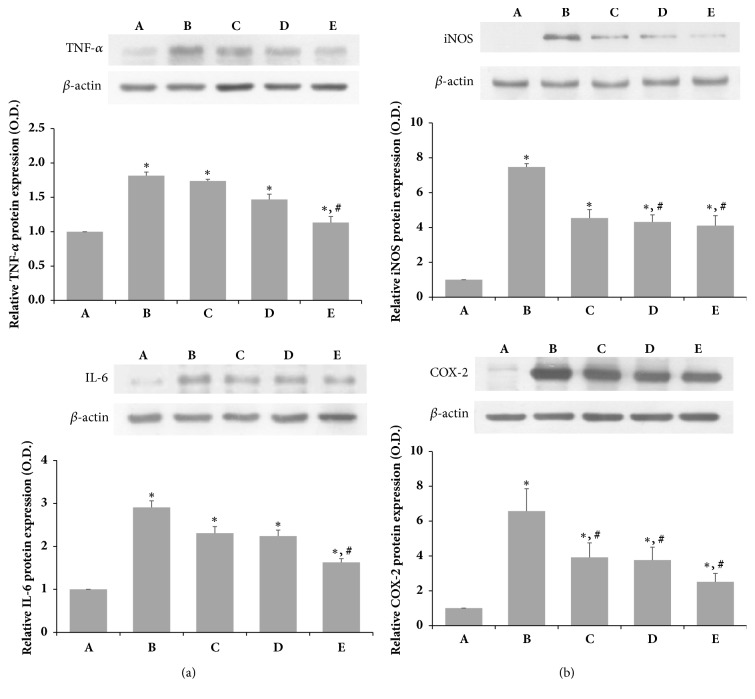
*Effects of SSM on LPS-induced protein expression of TNF-α, IL-6, iNOS, and COX-2*. RAW 264.7 cells were induced with 1 *μ*g/mL LPS and various concentrations of SSM for 24 h. (a) Relative protein expression levels of TNF-*α* and IL-6. (b) Relative protein expression levels of iNOS and COX-2. Bands were detected using an enhanced chemiluminescence (ECL) detection kit. Actin was used as an internal control (46 kDa). (A) Control group, (B) 1 *μ*g/mL LPS-administered group, (C) 1 *μ*g/mL LPS-administered and 0.1 *μ*g/mL SSM-treated group, (D) 1 *μ*g/mL LPS-administered and 1 *μ*g/mL SSM-treated group, and (E) 1 *μ*g/mL LPS-administered and 10 *μ*g/mL SSM-treated group. The results are presented as the means ± standard errors of the mean (SEMs). ^*∗*^P<0.05 compared to the control group. ^#^P<0.05 compared to the LPS-induced group.

The relative levels of TNF-*α*, IL-6, iNOS, and COX-2 were determined to confirm the effects of SSM on the protein levels of proinflammatory cytokines and inflammatory-related factors (Figures 3(a) and 3(b)). LPS administration increased expression of the inflammatory mediators TNF-*α*, IL-6, iNOS, and COX-2. However, SSM treatment decreased the levels of proinflammatory cytokines, such as TNF-*α* and IL-6, in a dose-dependent manner (Figure 3(a)). Furthermore, it significantly inhibited iNOS and COX-2 expression under LPS-induced inflammatory conditions in a dose-dependent manner (Figure 3(b)).

### 3.3. Effects of SSM on LPS-Induced Protein Expression of NF-*κ*B and I*κ*B*α*

**Figure 4:**
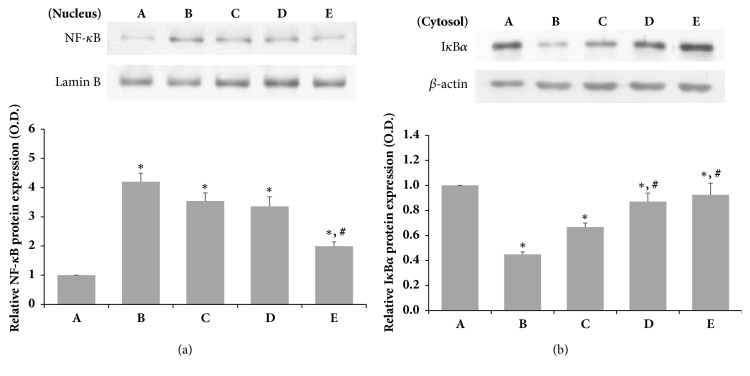
*Effects of SSM on LPS-induced NF-κB translocation and degradation of IκBα protein expression*. The cells were induced with 1 *μ*g/mL LPS and various concentrations of SSM for 24 h. (a) Relative protein expression of NF-*κ*B in the nucleus. (b) Relative protein expression of I*κ*B*α* in the cytosol. Bands were detected using an enhanced chemiluminescence (ECL) detection kit. Actin was used as an internal control (46 kDa). (A) Control group, (B) 1 *μ*g/mL LPS-administered group, (C) 1 *μ*g/mL LPS-administered and 0.1 *μ*g/mL SSM-treated group, (D) 1 *μ*g/mL LPS-administered and 1 *μ*g/mL SSM-treated group, and (E) 1 *μ*g/mL LPS-administered and 10 *μ*g/mL SSM-treated group. The results are presented as the means ± standard errors of the mean (SEMs). ^*∗*^P<0.05 compared to the control group. ^#^P<0.05 compared to the LPS-induced group.

As shown in Figure 4(a), we obtained the cytosolic fraction and nuclear protein fraction to investigate whether the expression of NF-*κ*B was due to decreased I*κ*B*α* protein expression. The protein level of NF-*κ*B in the group treated with SSM after LPS induction was decreased at high SSM concentrations. In addition, the cytosolic level of IкB*α* protein expression was reduced by LPS but was increased by treatment with SSM at high concentrations (Figure 4(b)). These results indicate that SSM treatment significantly increases I*κ*B*α* protein expression and NF-*κ*B protein expression in LPS-induced cells.

### 3.4. Effects of SSM on SFI and Thermal Hyperalgesia of SCNI in Rats

**Figure 5:**
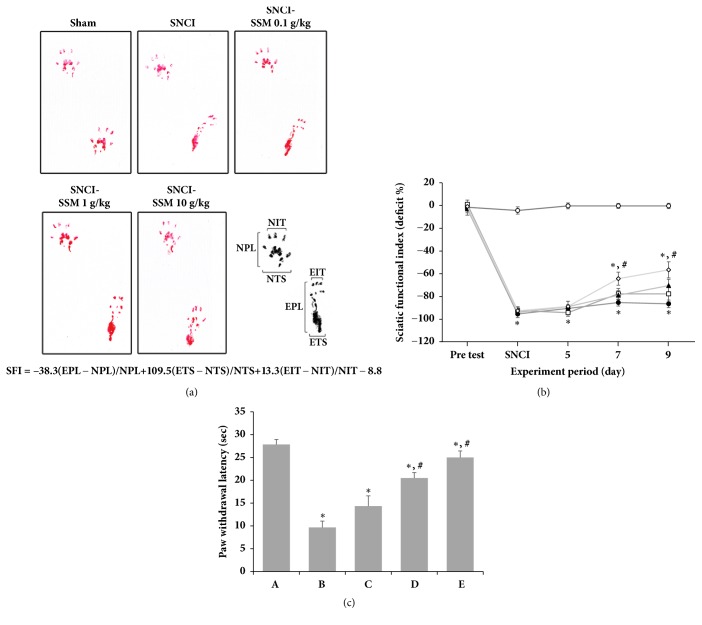
*Effects of SSM treatment on sciatic functional index (SFI) and paw withdrawal latency (PWL) following sciatic nerve crush injury*. (a) Walking track footprints in SNCI rats. (b) Plot showing the time-dependent changes in SFI value in each group. (○) Sham operation group, (●) SNCI-induced group, (□) SNCI-induced and 0.1 g/kg SSM-treated group, (▲) SNCI-induced and 1 g/kg SSM-treated group, and (*◇*) SNCI-induced and 10 g/kg SSM-treated group. (c) The PWL in the plantar test in each group. (A) Sham operation group, (B) SNCI-induced group, (C) SNCI-induced and 0.1 g/kg SSM-treated group, (D) SNCI-induced and 1 g/kg SSM-treated group, and (E) SNCI-induced and 10 g/kg SSM-treated group. The results are presented as the means ± standard errors of the mean (SEMs). ^*∗*^P<0.05 compared to the sham operation group. ^#^P<0.05 compared to the SNCI-induced group.

The SFI values in the walking track analyses are shown in Figures 5(a) and 5(b). The mean SFI for each group was calculated on day 1 before and 1, 5, 7, and 9 after surgery. The values in the sham operation group remained around 0 to -10 throughout the duration of the experiment. After surgery, the values of all operation groups dropped to close to -100 and changed slowly throughout the experiment. However, in SSM-treated groups, the values increased beginning at 7 days after surgery in a dose-dependent manner. On day 9 after surgery, the 10 g/kg SSM-treated group showed significantly better recovery. These results indicate that SSM treatment promotes functional locomotor recovery following SNCI. Thermal hyperalgesia was measured using the plantar test and is represented as the paw withdrawal latency (PWL) (Figure 5(c)). Induction of SNCI significantly decreased the PWL compared to the sham operation group. However, SSM treatment significantly increased PWL in a dose-dependent manner. These observations indicate that SSM treatment can alleviate the pain induced by SNCI and that a high dose may be the most effective to relieve neuropathic pain.

### 3.5. Effects of SSM on NF-200 Expression Following SNCI in Rats

**Figure 6:**
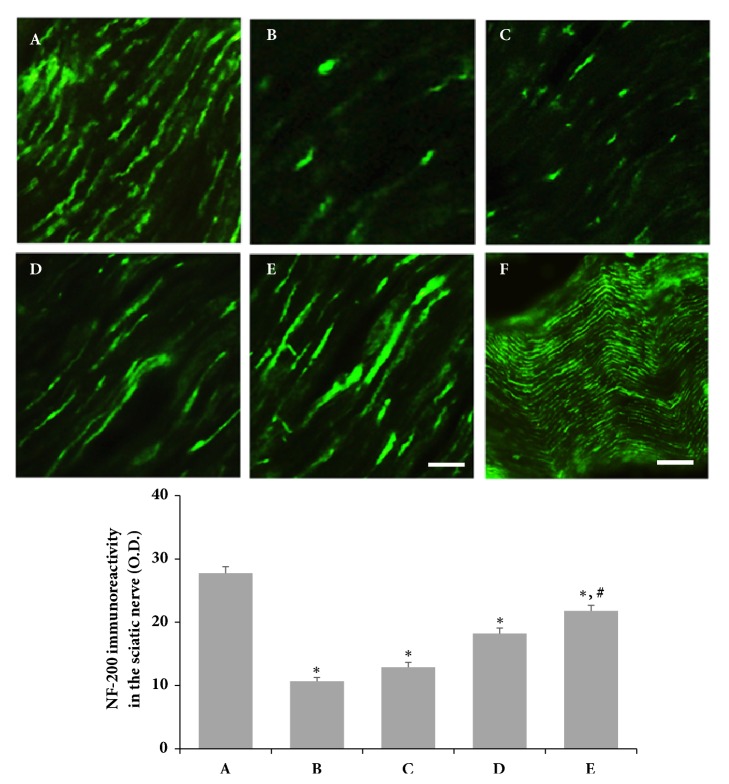
*Effects of SSM treatment on neurofilament (NF-200) expression in the sciatic nerve following sciatic nerve crushed injury*. Upper: photomicrographs of NF-200-immunoreactive fibers in the sciatic nerve. (A) Sham operation group, (B) SNCI-induced group, (C) SNCI-induced and 0.1 g/kg SSM-treated group, (D) SNCI-induced and 1 g/kg SSM-treated group, and (E) SNCI-induced and 10 g/kg SSM-treated group. (F) Whole nerve. The scale bars represent 400 *μ*m (A-E) and 100 *μ*m (F). Lower: optical density of NF-200 expression in each group. The results are presented as the means ± standard errors of the mean (SEMs). ^*∗*^P<0.05 compared to the sham operation group. ^#^P<0.05 compared to the SNCI-induced group.

Photomicrographs of NF-200-immunoreactive fibers in the sciatic crushed nerve are shown in Figure 6. SNCI significantly decreased NF-200 expression at the site of nerve crush compared to the sham operation group. However, SSM treatment enhanced the reduction of NF-200 expression induced by SNCI in a dose-dependent manner.

### 3.6. Effects of SSM on TNF-*α*, IL-6, iNOS, and COX-2 Expression Following SNCI in Rats

**Figure 7:**
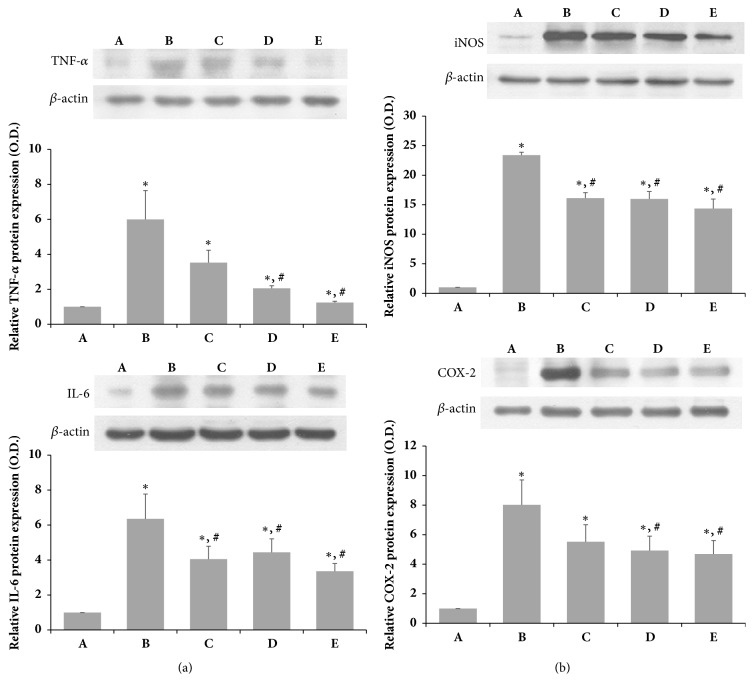
*Effects of SSM on SNCI-induced protein expression of TNF-α, IL-6, iNOS, and COX-2*. The sciatic nerve was crushed for 30 s using a surgical clip. (a) Relative protein expression levels of TNF-*α* and IL-6. (b) Relative protein expression levels of iNOS and COX-2. Bands were detected using an enhanced chemiluminescence (ECL) detection kit. Actin was used as an internal control (46 kDa). (A) Sham operation group, (B) SNCI-induced group, (C) SNCI-induced and 0.1 g/kg SSM-treated group, (D) SNCI-induced and 1 g/kg SSM-treated group, and (E) SNCI-induced and 10 g/kg SSM-treated group. The results are presented as the means ± standard errors of the mean (SEMs). ^*∗*^P<0.05 compared to the sham operation group. ^#^P<0.05 compared to the SNCI-induced group.

To determine the effects of SSM on inflammatory mediators related to pain, the levels of TNF-*α*, IL-6, iNOS, and COX-2 expression in the crushed sciatic nerve were examined as shown in Figures 7(a) and 7(b). SNCI markedly increased the levels of proinflammatory cytokines such as TNF-*α* and IL-6 at the site of injury in the sciatic nerve compared to the sham operation group (Figure 7(a)). In addition, the expression levels of inflammation-related factors iNOS and COX-2 were also increased by SNCI (Figure 7(b)). On the other hand, the levels of TNF-*α* and IL-6 expression in the injury site were significantly suppressed only at 10 g/kg SSM. SSM treatment also significantly inhibited iNOS and COX-2 expression at the site of injury in the sciatic nerve in a dose-dependent manner.

## 4. Discussion

The pathogenesis of nociceptive and inflammatory reactions are complex and multifactorial and are triggered and maintained by various intracellular mediators [21]. Among them, NO is a critical mediator and controller of inflammatory processes and is produced at high levels during inflammation. Overexpressed NO reacts with superoxide, leading to tissue damage, and causes pathological development of chronic inflammatory disorders [22, 23]. One of the other inflammatory mediators, PGE_2_, is a key inflammatory mediator, which is mainly synthesized by COX-2 in a variety of tissues under inflammation-related pathological conditions [24]. PGE_2_ is also a pain-inducing factor, which sensitizes primary sensory neurons, generates central sensitization, and facilitates the release of pain-related neuropeptides [23, 25]. PGE_2_ is formed during catalytic reactions to COX-2, and COX-2 can be produced by sequential production of NO and iNOS [26]. Some experiments showed that increase in COX-2 plays an important role in inducing the production of PGE_2_ and is closely related to the occurrence of neuropathic pain [27, 28]. Our experiments showed that NO and PGE_2_ concentrations were increased when the inflammatory response of the cultured cells was induced by LPS and significantly decreased by SSM treatment. Furthermore, SSM treatment of inflammatory cells and SNCI-induced animal models resulted in significant reductions in the elevated expression of iNOS and COX-2. Thus, the regulation of iNOS and COX-2 expression after SSM treatment may play a role in regulating the reduction of NO and PGE_2_. In previous studies, experiments involving inflammation of SNCI have indicated that PGE_2_ is associated with COX-2 [24]. Zhao et al. [29] suggested that SSM inhibited mRNA expression and arachidonic acid-metabolizing COX-2, CYP4A, and PGE_2_ production in tumor-associated macrophages. Some experiments reported that quinoline alkaloids and 2,4-di-tert-butylphenol among the SSM components have been reported to have potent antioxidative effects [30] and exhibited antiatherosclerotic effects in rats by controlling lipid peroxidation and NO by regulating the endothelin-1 system [31].

Numerous experimental studies have provided evidence that proinflammatory cytokines induce or facilitate inflammation as well as neuropathic pain and hyperalgesia. Previous report suggested that neuropathic pain induced by chronic constriction injury was associated with upregulation of sciatic TNF-*α* and IL-6 expression [32, 33]. Moreover, previous experiments reported that anti-inflammatory cytokine activation and proinflammatory cytokine inactivation are associated with antiallodynic effects in a rat model of neuropathic pain [34]. The present study showed that increased levels of TNF-*α* and IL-6 induced by LPS stimulation were reduced by SSM treatment* in vitro*. In addition, increases in levels of TNF-*α* and IL-6 expression by SNCI were reduced by SSM treatment in the rat. Several studies have suggested that Arg^561^-Val^562^ peptide binding of SSM component may be effective for inducing fibrinolytic activity or anti-inflammatory effects [9, 35].* Scolopendra* extracts increase the levels of IL-2, IL-4, and IL-10 expression, effectively alleviating the pain associated with joint damage [12]. Furthermore,* Scolopendra* and* Scorpio* treatment downregulates TNF-*α*, IL-1*β*, IL-4, and IL-10 expression via T lymphocyte regulation in patients with rheumatic arthritis [17]. We confirmed that LPS stimulated cells and led to changes in inflammatory factors and experimentally confirmed that SSM treatment affected I*κ*B*α* and NF-*κ*B expression in the nucleus and cytoplasm. NF-*κ*B is a factor that is produced rapidly by cell injury stimulation, including exposure to ROS, TNF-*α*, IL-1*β*, LPS, and radiation. Activation of NF-*κ*B is initiated by signal-induced degradation of the I*κ*B protein, and the activated NF-*κ*B complex translocates from the cytoplasm to the nucleus, which can produce transcription factors such as TNF-*α*, IL-1*β*, and IL-6 [36–38]. Our data present that activation of IкB*α* was significantly reduced by SSM treatment following LPS damage in RAW 264.7 cells, and enhanced NF-*κ*B protein expression was significantly decreased by SSM treatment. These results suggest that SSM may inhibit NF-кB activation by attenuating the phosphorylation of I*κ*B*α* and the translocation of the p50 and p65 subunits of NF-*κ*B from the cytosol to the nucleus in LPS-induced RAW 264.7 cells. A previous study suggested that SSM inhibited the activation of c-Jun NH2-terminal kinase, p38, and NF-*κ*B in pancreatitis by inhibiting HMGB-1 [16]. Thus, SSM treatment may reduce inflammatory factors via the NF-*κ*B pathway in cells as well as in animal models. Clinically, peripheral nerve damage is characterized by neuropathic pain and behavioral disturbances. Sciatic nerve ligation of rats is the most commonly used model for neuropathic studies because it can easily be reproduced and is similar to human neuropathy due to peripheral nerve trauma [39]. In particular, peripheral nerve impairment induces hyperalgesia and allodynia. In fact, peripheral nerve injury causes hyperalgesia and thermal hyperalgesia in SNCI rats [1]. In the present study, we examined neurological and behavioral changes associated with neurological damage in SNCI models treated with SSM. Our results showed that locomotor activity due to nerve damage is improved by treatment with SSM in SFI tests. Some reports showed that treatment with* Scolopendra *mixture extract improves exercise capacity in a rat collagen-induced arthritis joint injury model [12]. Moreover, SSM treatment reduced spinal nerve ligation-induced mechanical allodynia and thermal hyperalgesia through inactivation of microglia and astroglia in animal model [40]. In the present study, thermal hyperalgesia tests were performed to evaluate the effects of SSM on allodynia and hyperalgesia caused by peripheral nerve damage in a SNCI model. The withdrawal delay was improved in animals treated with SSM repeatedly compared to animals induced only with SNCI. Therefore, the withdrawal delay of SNCI-induced animals seemed to be improved by repeated SSM treatment. The results presented here suggest that SSM treatment may be effective for restoring sensory function and motor ability in animals following sciatic nerve injury through inhibition of inflammation. Interestingly, confirming the histological changes in the neurofilament, we found that the SSM-treated group showed a greater nerve regeneration effect than the SNCI-induced group. A previous study showed that SSM treatment attenuates the loss of motor neurons in the spinal cord of symptomatic hSOD1^G93A^ transgenic mice [41]. Therefore, SSM treatment seems to improve locomotor activity and sensory nerve function as well as nerve regeneration in SNCI.

## 5. Conclusions

The SSM treatment modulates inflammatory factors, such as iNOS, TNF-*α*, and IL-6, and regulates pain-related factors, such as PGE_2_ and COX-2, by regulating activity through I*κ*B*α* and NF-*κ*B within the cell. Therefore, treatment with SSM in neuropathic lesions appears to have a beneficial effect on neuropathic pain by modulating inflammatory factors and improving hyperalgesia and locomotor performance. Based on these experimental results, SSM is expected to be an effective drug candidate for treatment of peripheral nervous system diseases, such as SNCI.

## Data Availability

The data used to support the findings of this study are available from the corresponding author upon request.
